# Two-Dimensional and Three-Dimensional Single Particle Tracking of Upconverting Nanoparticles in Living Cells

**DOI:** 10.3390/ijms20061424

**Published:** 2019-03-21

**Authors:** Kyujin Shin, Yo Han Song, Yeongchang Goh, Kang Taek Lee

**Affiliations:** Department of Chemistry, School of Physics and Chemistry, Gwangju Institute of Science and Technology (GIST), Gwangju 61005, Korea; shinkj@gist.ac.kr (K.S.); yhsong@gist.ac.kr (Y.H.S.); kyc940409@gist.ac.kr (Y.G.)

**Keywords:** upconversion nanoparticles, single-particle tracking, three-dimensional imaging, intracellular transport

## Abstract

Lanthanide-doped upconversion nanoparticles (UCNPs) are inorganic nanomaterials in which the lanthanide cations embedded in the host matrix can convert incident near-infrared light to visible or ultraviolet light. These particles are often used for long-term and real-time imaging because they are extremely stable even when subjected to continuous irradiation for a long time. It is now possible to image their movement at the single particle level with a scale of a few nanometers and track their trajectories as a function of time with a scale of a few microseconds. Such UCNP-based single-particle tracking (SPT) technology provides information about the intracellular structures and dynamics in living cells. Thus far, most imaging techniques have been built on fluorescence microscopic techniques (epifluorescence, total internal reflection, etc.). However, two-dimensional (2D) images obtained using these techniques are limited in only being able to visualize those on the focal planes of the objective lens. On the contrary, if three-dimensional (3D) structures and dynamics are known, deeper insights into the biology of the thick cells and tissues can be obtained. In this review, we introduce the status of the fluorescence imaging techniques, discuss the mathematical description of SPT, and outline the past few studies using UCNPs as imaging probes or biologically functionalized carriers.

## 1. Introduction

**Figure 1:**
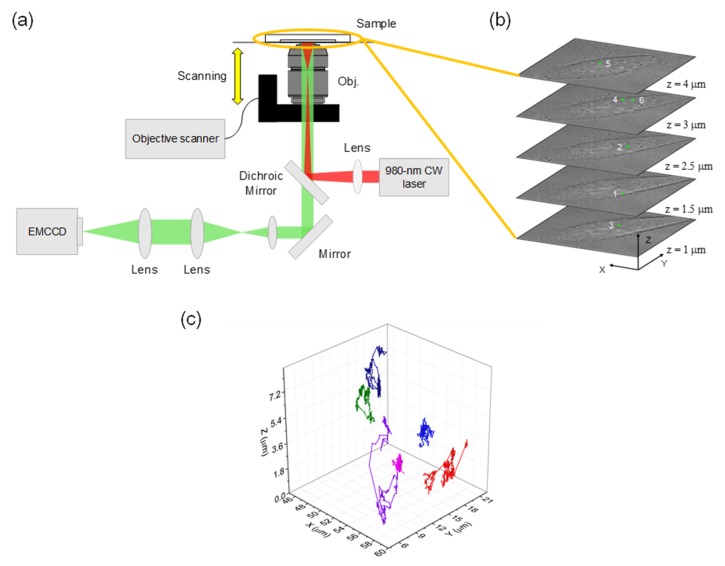
(**a**) Wide-field epi-fluorescence microscopy setup for 3D imaging and tracking. (**b**) Five representative single-cell images containing UCNPs at different heights. (**c**) 3D trajectories of six single UCNP vesicles.

The synthesis and characterization of lanthanide-doped upconversion nanoparticles (UCNPs) are well established [1,2,3,4,5,6]. In particular, UCNPs, which are doubly doped by the sensitizer cations (usually Yb^3+^) and the activator cations (e.g., Yb^3+^, Er^3+^, Tm^3+^, Nd^3+^, Ho^3+^) in the host material (e.g., NaYF_4_, NaGdF_4_), have attracted the greatest interest [4,5]. By selecting a proper cation pair and adjusting their relative composition, their optical properties can be optimized, such as wavelengths, band-width, quantum yield, and photo stability [1,4]. UCNPs are excited by near-infrared (NIR) lasers, whose wavelength is well matched to the absorption wavelength of Yb^3+^ (980 nm) and Nd^3+^ (808 nm) [1,2,3,4,5]. In this NIR range, biomolecules show minimum absorption in vitro and in vivo [7,8,9,10,11,12]. As a result, they do not damage the biological samples (cells and tissues) optically and chemically. Thus, UCNPs can be monitored for a long time [13,14]. In addition, for the same reason, the autofluorescence is nearly absent, while this side effect is problematic even in confocal microscopy [15,16]. Finally, NIR can penetrate into the tissues and skins as deep as a few cm due to the lower scattering and absorption compared to visible or UV light, which would be beneficial when UCNPs are employed in medical applications [17,18,19,20]. Other than the benefits due to the NIR excitation, the upconversion process itself confers huge advantages. Their emission upon continuous irradiation of NIR was found to be free of blinking and bleaching [21,22,23]. Thus, UCNPs combined with NIR excitation will help researchers to receive successful long-term and real-time images or movies [13,14,16,24]. Being free from autofluorescence, these movies provide the accurate coordinates of UCNPs as a function of time. The duration of such “tracking” of particles and the length of trajectories are obviously connected to the photostability of the probes (organic dyes, fluorescence proteins, and nanoparticles) [25,26,27,28]. In that respect, UCNPs are considered ideal for optical imaging due to their photostability, as described above [3,4]. However, the data analysis for determining the coordinates is not trivial because one has to obtain the images of individual particle signals that are well separated spatially without significant overlap. In order to deal with such a situation, several versions of super-resolution fluorescence microscopy using UCNPs have been recently developed and are widely used [23,29,30,31,32]. In this review, we introduce the status of the fluorescence imaging techniques with an emphasis on the benefits of three-dimensional (3D) imaging over the conventional two-dimensional (2D) version (and b). After this, we discuss the mathematical description of SPT (Figure 1c) and the past few studies, using UCNPs as imaging probes or biologically functionalized carriers.

## 2. Single-Particle Tracking

### 2.1. Principle

In biological science studies, SPT may be significantly more informative than any other methods [25,26,27,33]. Literally, SPT analysis tracks the position of single particles (e.g., a single nanoparticle, vesicle, or endosome). To construct the trajectories of single particles, the center position of particle is connected by a tracking algorithm [25,26,27]. It is possible to obtain various quantitative analyses about the motion by using the obtained trajectories. As the imaging probe, UCNPs might be compared to semiconductor quantum dots (QDs) in many ways. For example, the absorption wavelength of QDs is spectrally broad while the emission is narrow, and its wavelength depends strongly on the size (the smaller the bluer) due to the quantum confinement effect [34,35,36]. On the contrary, UCNPs are usually excited at a single wavelength (980 or 808 nm) and their emission is blue-shifted (i.e., towards higher frequencies) [1,2]. It is important to note that the spectra of UCNPs do not depend on their size, which indicates that any variations in the particle dimensions (usually from sub-10 nm to several hundred nm) do not affect their photophysical properties [37,38].

### 2.2. Localization of Single Particles

**Figure 2:**
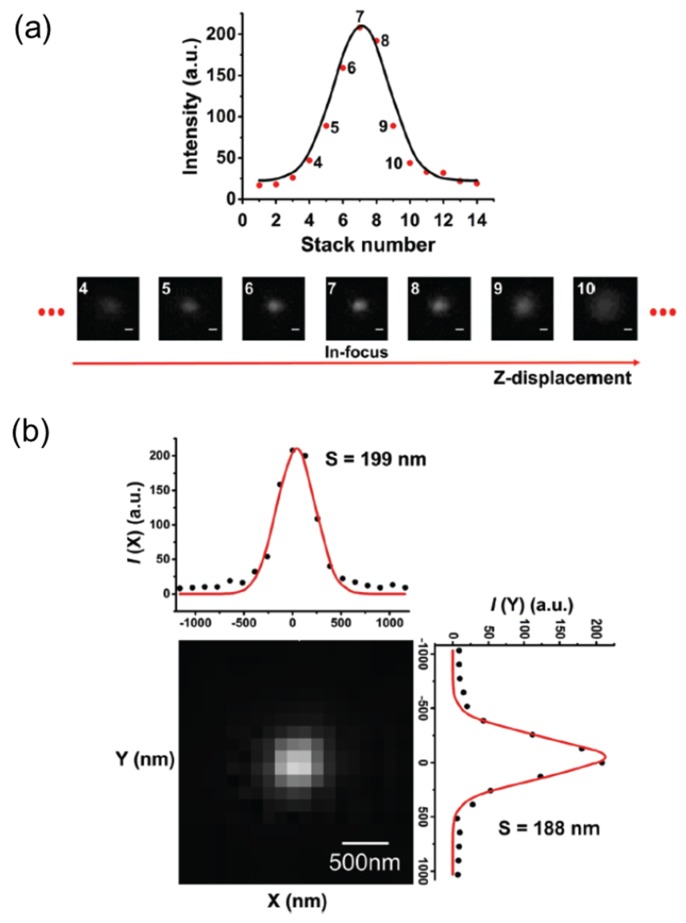
(**a**) The UCNP intensity profile over the *z*-axis and the Gaussian fit. UCNP images were acquired by 980 nm excitation (731 W cm^−2^) with an exposure time of 70 millisecond. Scale bar: 500 nm. (**b**) A typical 3D centroid determination. The UCNP on the section image that is closest to the z-coordinate determined in (**a**) is chosen for the localization. The numbers in the intensity plots represent standard deviations of the Gaussian function [16]. Reproduced by permission of the PCCP Owner Societies. Copyright 2018 Royal Society of Chemistry.

To track a single fluorophore, its coordinates need to be accurately determined. If we assume that the fluorophore is a point source of the photon emission, the real image is a large spot due to the diffraction limit of light. This spot signals display the shape of a function. This function, which is known as the “point-spread function (PSF),” is defined as the optical instrument response function of the microscope. In a typical experimental setup, such PSFs are isotropic and contain the information about the coordinates [27,39,40]. On the focal plane, a PSF is fit to a 2D Gaussian functions, by which the x- and y-coordinates are determined (Figure 2b) [39]. Although the Airy function may be the best choice as a model PSF, most localization studies adopt the Gaussian function (Equation (1)) instead of the Airy function (Equation (2)) because the Gaussian function is much easier to handle and the localized centroids are accurate enough [27].

(1) $$\begin{array}{c} \text{Gaussian for x-y plane}\equiv A\cdot \exp(-\frac{{\left(x-x_{0}\right)}^{2}+{\left(y-y_{0}\right)}^{2}}{2\sigma^{2}})\\ A:\;\text{amplitude of Gaussian},\;\sigma :\;\text{standard deviation of Gaussian} \end{array}$$

(2) $$\begin{array}{c} \text{Airy function}\equiv {\left(\frac{2J_{1}(\frac{2\pi }{\lambda }NA\sqrt{{\left(x-x_{0}\right)}^{2}+{\left(y-y_{0}\right)}^{2}})}{\frac{2\pi }{\lambda }NA\sqrt{{\left(x-x_{0}\right)}^{2}+{\left(y-y_{0}\right)}^{2}}}\right)}^{2}\\ J_{1}(X):\;\text{the first order Bessel function},\;\lambda :\;\text{wavelength of emission},\;NA:\;\text{numerical aperture} \end{array}$$

The center, which is the point where the Gaussian function is maximum, is defined as two- dimensional x- and y- coordinates [27,40]. The accuracy of localization can be estimated by the following equation [39]:

(3) $$∆x=\sqrt{\frac{\sigma^{2}+a^{2}/12}{N}+\frac{8\pi \sigma^{4}b^{2}}{a^{2}N^{2}}}\approx \sqrt{\frac{\sigma^{2}+a^{2}/12}{N}},$$

where σ is the standard deviation of the fitting Gaussian function, a is the pixel size, b is the background noise, and N is the photon number. In practice, the second term is negligibly small [39]. In this equation, it is noteworthy that the accuracy is closely related to the number of photons detected. The s and a values depend on the optical setup and the camera technology, so these cannot be controlled [39]. However, the number of photons (N) is the number related to the intrinsic stability of the fluorophore against irradiation or excitation [27,39,40]. A high N value can be achieved by raising the power of the light source (laser). However, the fluorophores would become photobleached and disappear from the images more frequently. This is why people are concerned about the photostability or quantum yield of their probes (organic dyes) [23,27,28]. What about UCNPs? The quantum yield of UCNPs is generally very low (<1%) compared to organic dyes or QDs [1,2]. However, the laser power can be raised until any saturation occurs [31]. The signal will increase accordingly, with only small damage to the cells and tissues [10,13,14].

### 2.3. Two-Dimensional Single-Particle Tracking

Most SPT algorithms are built on 2D localization, whereby the center positions of the particles are determined in every frame of the movie. Various SPT algorithms have been developed, from “nearest neighbor algorithms” to more sophisticated custom algorithms, which have been reviewed previously [27,41,42]. In so doing, the research points are focused on how to identify the same particles in the subsequent frames. Chenouard et al. compared the performance of 14 different SPT algorithms for different particle dynamics (random diffusive motion, directed motion, and their combination) and concluded that no single SPT algorithm is optimal for all situations [42]. Recently, the theoretical research groups studying nonlinear statistical mechanics suggested that these mechanics may be helpful for the analysis of the trajectory in terms of velocity distribution, cumulative displacement, mean square displacement (MSD), non-Gaussian parameter (NGP) and ergodicity breaking parameter analyses [26,27,43,44,45].

Because UCNPs do not exhibit photoblinking, their continuous movements can be monitored without interruption as long as the particles are close to the focal plane (2D space) [13,14]. Thus, for cells with a finite thickness, the movements along the *z*-axis are lost and only the particles moving on the *x*- and *y*-axis can be investigated further [13,14,33]. To be frank, the particles that people have been looking at are moving by accident along the focal plane. Even the absence of photoblinking of UCNPs cannot overcome this problem [14]. However, it can be distinguished whether the UCNPs that “fade away” or “show up” in the movies are moving out of focus or in focus, respectively. In this respect, there is no way to tell which is which due to the extensive blinking of QDs. This is one of the disadvantages of 2D trajectories.

However, even with 2D trajectories, one may obtain substantial insights into the cellular dynamics [14,26,33,43,46,47,48,49,50]. One of the representative types of dynamics is transport by the motor proteins. Motor proteins running on the microtubule (dyneins and kinesins) or actin filament (myosin) play important roles for intracellular transport and as part of the cytoskeleton [51,52]. The consensus is that the internalization of nanomaterials occurs through a process called “endocytosis” [13,53,54,55,56,57]. The nanoparticles are captured by the motor proteins and transported through directional motion (anterograde, retrograde and/or bidirectional) along the microtubule and some fraction of the nanoparticles are released by exocytosis [13,56,57]. Such endocytic pathways form the basic concept of the drug and gene delivery [58,59,60,61,62,63,64]. These steps are oversimplified and the uptake rate may be stochastic or highly controlled [56,57,65,66,67]. The presence of multimode intracellular transport in living cells was demonstrated using long-term SPT with UCNPs [14]. Through a real-time imaging study of UCNPs in living cells, Nam et al. tracked the movement of vesicles containing UCNPs in living HeLa cells and obtained a real-time image of endocytosed UCNPs at the single vesicle level for 6 h [14]. The 2D SPT analysis demonstrated that the dynamics of particle transport was composed of multiple phases within a single trajectory, including active transport by motor proteins, such as dyneins and kinesins [52].

### 2.4. Three-Dimensional Localization and Single Particle Tracking

As most biological samples are 3D objects, 2D SPT has limitations, especially when one studies thick cells or tissues. The 3D localization is much more challenging than 2D localization because the 3D PSF is axially symmetric with respect to the focal plane and the shape changes slowly as the axial position varies [40]. Thus far, a number of 3D optical imaging techniques have been developed [68,69,70,71,72,73,74,75,76,77,78,79,80]. In all techniques, the researchers scan their illumination over the sample to complete 3D images [68,69,70]. All types of this scanning method have a vertical directional component, *z*, with respect to the 2D images being obtained. For example, in epifluorescence microscopy, the scanning direction of the objective lens and/or the sample stage is perpendicular to the 2D images. On the contrary, if the angle between the exciting light (e.g., lasers) and aqueous environment reaches the critical angle (~60°), total internal reflection (TIR) starts to occur and a thin evanescent field that is less than ~200 nm deep is generated on the interface [81,82]. Total internal reflection fluorescence (TIRF) microscopy can only be used to excite fluorophores within the field. Therefore, TIRF is not generally appropriate for the study of the cells and tissues thicker than ~200 nm.

There are various types of 3D microscopy, which use scattering or fluorescence signals for detection [69,70,71,72,73,74,75,76,77,78,79,80]. It is noteworthy that scattering microscopies were used for the 3D particle tracking. Among the various techniques, digital holographic microscopy (DHM) or interferometric scattering microscopy (iSCAT) are non-destructive “label-free” methods [71,72,73,74]. They have been developed using nonlinear scattering of light instead of using imaging probes. These are really smart and powerful methods but the focus here will be placed on the 3D SPT of UCNPs, which is applicable for observing subcellular dynamics in living cells. Previous studies have demonstrated 3D localization methods using engineered 3D PSF (e.g., double-helix PSF with fluorescence of single dyes), bifocal imaging scheme or multiplexed illumination (e.g., TSUNAMI), which is an optical aberration to understand dynamics in living cells at the single molecule level [75,76,77,78,79,80].

In astigmatism, 3D localization is a fully wide-field scheme, which is useful when the sample thickness along the *z*-axis is relatively small [78,79]. Zhuang and co-workers used the elliptical Gaussian (Equation (4)) and Airy-beam-based self-bending PSF instead of using Gaussian to fit the PSF shapes on the x–y plane [78,80,83].

(4) $$\text{Elliptical Gaussian}\equiv D\cdot \exp\left(-2{\left(\frac{x-x_{0}}{w_{x}}\right)}^{2}-2{\left(\frac{y-y_{0}}{w_{y}}\right)}^{2}\right)+bg,$$

where *D* is the amplitude of peak, (*x*_0_, *y*_0_) is the center of the emitter and *bg* is the background.

(5) $$\text{Image width},\;w_{x,z}(z)=w_{0}\sqrt{1+{\left(\frac{z-z_{0}}{d}\right)}^{2}+B{\left(\frac{z-z_{0}}{d}\right)}^{3}+C{\left(\frac{z-z_{0}}{d}\right)}^{4}},$$

where *w*_0_ is the width of the PSF at the focal plane, *z*_0_ is the offset of the x or y focal plane from the average focal plane, d is the depth of focus and *B* and *C* are correction coefficients. In this case, the lateral (x- and y-) and axial (z-) resolutions of 3D localization are ~30 and ~60 nm, respectively [78].

Due to UCNPs’ superior photostability and NIR excitation, combining 3D localization with UCNP will guarantee robust 3D tracking methodology [16,24]. However, the application of 3D localization methods using astigmatism and bifocal imaging is limited because the range of the z-position is relatively small (~500 nm) [76,78]. The 3D localization using multiplexed illumination microscopy requires a very sophisticated microscope [77]. On the other hand, the engineered PSF-based 3D localization, such as the double-helix PSF scheme, is regarded as a promising tool that is suitable for observing cellular dynamics by using UCNPs [75].

As noted above, the out-of-focus background due to autofluorescence is minimized, so 3D movement of all the particles can be monitored at the same time and, consequently, the location of UCNPs at the specific binding can be visualized [16,24]. The experimental approach to 3D localization of UCNPs can be categorized into two types of techniques: “z-scanning” and the “wide-field” scheme [16]. For 3D localization of UCNPs, the first step involves finding the focal plane and z-coordinate. To this end, z-scanning is performed using actuators, such as a stepping motor scanner or a piezoelectric scanner. Using a software code, the position of the objective or the sample holder on the microscope can be adjusted. While monitoring the emission intensity of a spot (Figure 2a), the distance between the objective lens and the sample are scanned. In order to find the focal plane, the UCNP intensity vs. frame number is plotted and fitted to Gaussian distribution. The use of Gaussian distribution as a function of z was inspired by the 2D SPT point-spread function (Equation (6)) but for the purpose of centroid determination, Gaussian is a good approximation [16].

(6) $$\begin{array}{c} \text{Gaussian for}z\text{-axis}\equiv A^{\prime }\cdot \exp(-\frac{{\left(z-z_{0}\right)}^{2}}{2{\sigma^{\prime }}^{2}})\\ A^{\prime }:\;\text{amplitude of Gaussian},\;\sigma^{\prime }:\;\text{standard deviation of Gaussian} \end{array}$$

We emphasize here that there are pros and cons to each of the methods, so it is important to understand all those schemes and select appropriate methods for the specific purpose. The technical aspects of the two schemes can be found in the literature and, therefore, the pros and cons of scanning microscopy (confocal) and wide-filed microscopy (epifluorescence microscopy and total internal reflection microscopy or TIRF) are summarized in Table 1.

**Figure 3:**
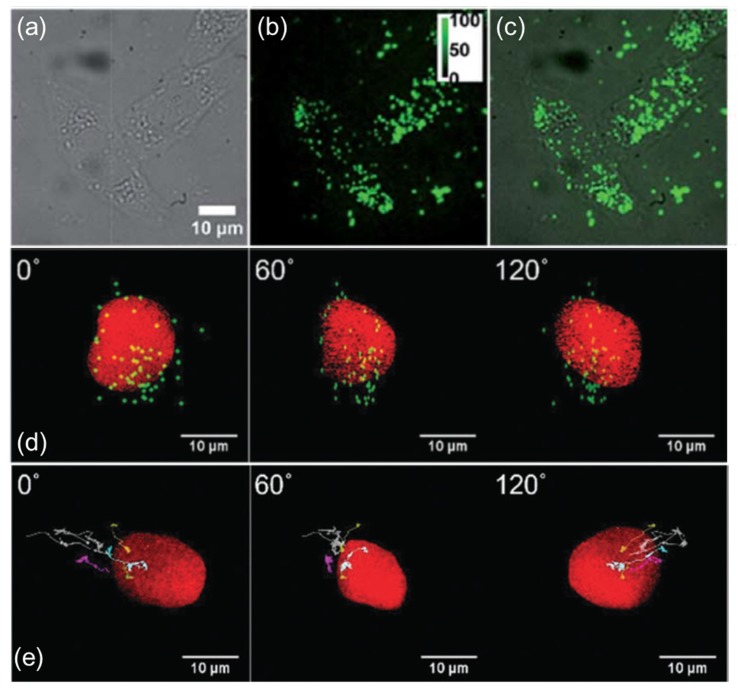
2D and 3D SPT images. (**a**–**c**) The fluorescence microscopy characterization of HeLa cells co-cultured with polymer-modified UCNPs. (**a**) bright-field, (**b**) fluorescence and (**c**) merged microscopy images [90]. (**d**) The images from various angles (at 0°, 60° and 120°) for UCNP–phospholipid–PEG-TAT. (**e**) The 3D trajectories from various angles (at 0°, 60° and 120°) for UCNP–phospholipid–PEG-NH^3+^ [45]. Reproduced from [90] with permission from Royal Society of Chemistry and [45] by permission of the PCCP Owner Societies. Copyright 2018 Royal Society of Chemistry.

It is easy to determine from Table 1 that these methods are complementary to each other and, as for the UCNP imaging, the epifluorescence microscopic technique was found to be the best [3]. For example, the high background stemming mainly from the solvent (medium) is simply removed by the rinsing solvent while preparing the immobilized cell samples. The 3D coordinates were determined upon reconstruction (Figure 3d,e) and the outline of nuclei whose surface is covered with red fluorescent protein (RFP) is shown. Since RFP is photobleached quickly, the snapshot pictures of nuclei are taken and the laser turned off (532 nm). Moreover, in 3D images, the interesting point is whether the particles are inside and outside the organelles, such as nuclei and the mitochondria cell membrane, addressing the mechanism of internalization and release. It is worth noting that this 3D experiment was a combination of wide-field and z-scanning. The imaging speed was one 3D image per second, thus useful for fast dynamics [16,45].

The method of confocal microscopy is based on a scanning scheme in all three (x-, y- and z-) directions that has been already reviewed in many studies [68,69,70]. Although this method is appropriate for watching fixed structures or slow dynamics, it is usually difficult to track fast moving particles. One of the breakthroughs to this was the spinning-disk confocal scheme, whereby the imaging speed can be substantially increased while preserving the advantages of confocal microscopy [84,85,86,87,88]. That is, the fast 2D sectioning and z-scanning capability of spinning-disk confocal microscopy are good alternative ways of tracking UCNPs in 3D.

## 3. Applications

The SPT of nanoparticles at the cellular level can be engineered for efficient drug or gene delivery and sensing [3,4,5,6,60,64,89]. For example, the information from SPT analysis provides the endocytosis, delivery pathway, and arrival at the target [14,57].

### 3.1. Delivery System

In drug delivery systems where nanoparticles are used as carriers, the most important factor is the number of nanoparticles that are efficiently taken up by the cell. Zhang et al. reported that UCNPs with a higher surface charge can be more efficiently taken up by the cell [90]. The uptake efficiency of UCNP-PEI (positively charged, PEI = polyethyleneimine) was higher than that of UCNP-PAA (negatively charged, PAA = polyacrylamide) or UCNP-PVP (weak positively charged, PVP = polyvinyl pyrrolidone) over the initial 4 h. This was attributed simply to the electrostatic interaction between nanoparticles and the negatively charged cell membrane, which was suggested to be the main driving force behind the controlled cellular uptake of nanoparticles (Figure 3a–c). In addition, as the particle size increases, the role of the surface charge weakens and the particle shape becomes a major determining factor in the cellular uptake process. Thus, particles with a large surface-to-volume ratio were more efficiently taken up in the energy-dependent endocytosis process.

In addition to cellular uptake in the delivery system, another important factor is precise targeting. Previous studies have shown that it is challenging to quantify the targeting efficiency at the cellular level as many nanoparticles are taken up by the cells and the whole image is analyzed. However, as shown in several previous studies, SPT of UCNPs in combination with target staining can allow sufficient analysis of the targeting accuracy [45,91,92,93,94,95].

Gho et al. evaluated the uptake efficiency and targeting effect of UCNPs with cell-penetrated peptides using the TAT peptide (cell penetration and nuclear-targeted sequences) conjugated with UCNP and UCNP-PAA [45]. The uptake efficiency of TAT-peptide-conjugated UCNP and UCNP-PAA did not differ significantly during the first 4 h. However, after 24 h, UCNP-TAT (which had a strong positive charge and was targeted to the cell nucleus) remains in the cells, whereas UCNP-PAA is mostly excreted outside the cells, indicating that its targeting effect was better than that of UCNP-PAA (Figure 3d). In particular, the real-time 3D tracking of UCNP using these methods can provide useful information regarding the intracellular pathways of UCNP in live cells. Simultaneously tracking several single UCNPs can allow the analysis of relative movement (Figure 3e). This technique is highly advantageous for studying the effect of various intracellular structures on carrier transport in delivery systems.

Wang et al. [94] studied the delivery of doxorubicin, an anticancer drug, using UCNP. They used folic-acid-conjugated UCNP to target folate receptors that are present on various types of cancer cell surfaces. They elucidated the exact targeting effect by tracking the location of doxorubicin and single UCNPs. Liu et al. [95] studied a photodynamic system that induced the apoptosis of cancer cells by targeting mitochondria and generating mitochondrial reactive oxygen species (mitoROS) through a photosensitizer-conjugated UCNP. In particular, they demonstrated that the heme-containing cytochrome c produced in this process could be used as an indicator for apoptosis studies by altering the upconversion luminescence signal. They stained the mitochondria with a fluorescent dye and tracked the location of a single UCNP to ensure that the correct target was reached.

### 3.2. Biosensing

In addition to drug delivery, SPT technology is actively used in biosensing. An acidic pH inside cells is an important indicator of intracellular dysfunction, such as cancer [96]. Therefore, the development of an effective pH sensor that is capable of instantly measuring and imaging rapid pH changes within cells would be an important breakthrough [96,97,98]. In particular, due to their photostability and use of NIR excitation, UCNP-based pH-sensitive probes can function as a sensor inside living cells for an extended period of time. Arppe et al. [96] developed a pH sensor that covalently linked a fluorogenic pH-dependent dye (pHrodo™ Red) to the surface of UCNP. However, the fluorescence emission of pHrodo™ Red significantly decreases with decreasing pH and cannot be used for long-term live cell imaging due to the phototoxicity of the 532 nm laser wavelength used to excite this dye, which shows photobleaching and photoblinking. However, pHrodo™ Red conjugated to UCNP was used as a stable sensor via the energy transfer effect. Hence, this group was able to develop a pH sensor with a dynamic pH range of 2.5–7.2 and demonstrated its function as a nanoscale pH sensor in living HeLa cells.

**Figure 4:**
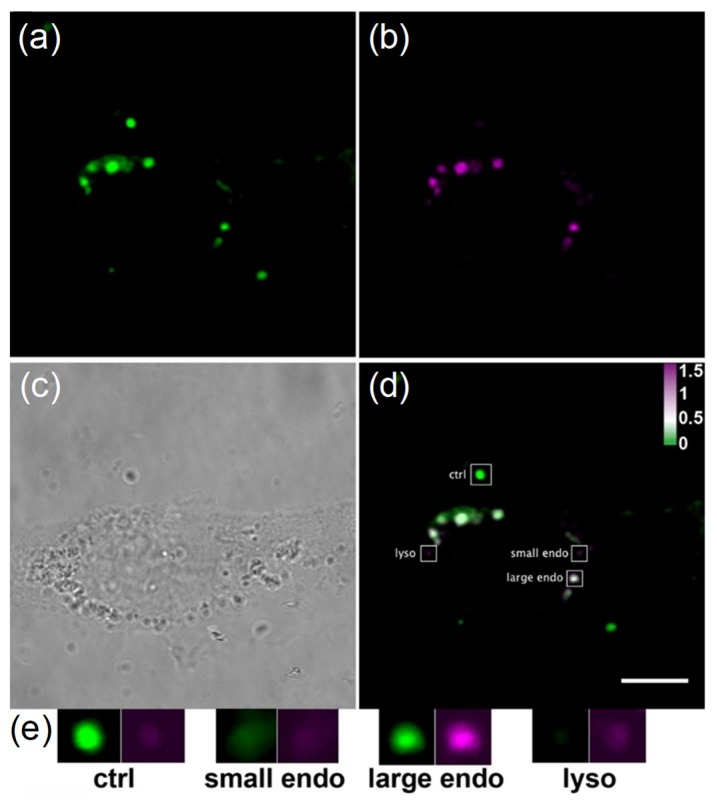
Ratiometric imaging of pH probes reveals their localization in three types of microenvironment. (**a**) Localization of UCNPs detected using 980 nm excitation, (**b**) sensitized upconversion resonance energy transfer emission from pHrodo Red, (**c**) bright-field, (**d**) an overlaid ratiometric image of pH nanoprobes with different ratios depending on the localization. Scale bar 10 μm. The enlarged insets (**e**) show different ratios in extracellular (ctrl), small endosome, large endosome, and lysosome. Brightness of insets is increased by 10 gray level units from the overlaid image (**d**) for better visibility [98]. Reprinted from [98] with permission from American Chemical Society. Copyright 2017 American Chemical Society.

Similarly, Näreoja et al. [98] used pHrodo conjugated to PEI-coated UCNP in a study of the regulation of intracellular vesicle acidity. Cells were treated with UCNP-PEI-pHrodo for 16 h. After this, the intracellular localized UCNP in the cells were monitored and the intracellular environment was classified into three categories, e.g., cytoplasmic (pH 7.2–7.5), endosomal (6.0–7.2) and lysosomal (<6.0). Most of the UCNPs (95%) were discharged from endosomes into the neutral environment that was located in lysosomes and endosomes. Thus, they could be used as sensors for measuring pH change inside the endoplasmic reticulum (Figure 4). These results would be used as a very useful tool for detecting the change in pH in a cellular level environment by imaging the position of the endoplasmic reticulum in real time. This would involve recording the change of the emission wavelength as a function of pH change at the single particle level.

**Figure 5:**
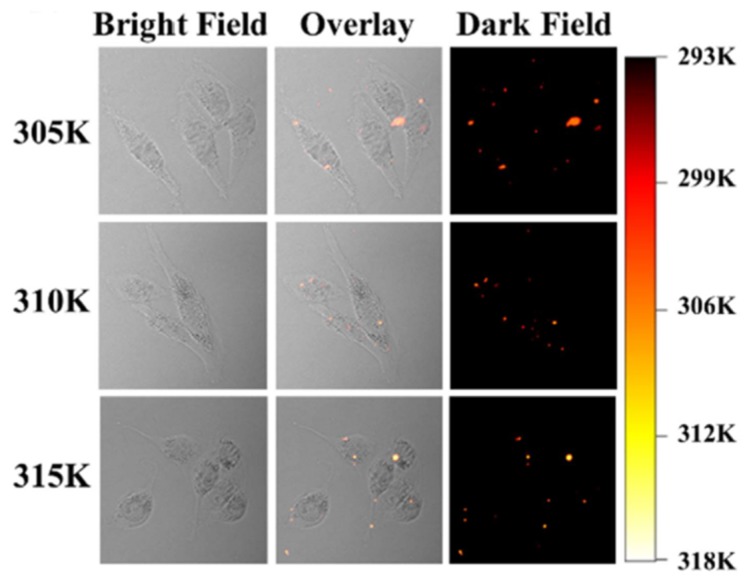
Thermal images of NIH-3T3 cells treated with external heating [100]. Adapted from [100] with permission from IOPScience. Copyright 2018 IOPScience.

Another important function of biosensors is heat sensing via thermosensor systems [99]. Thermal mapping requires fluorescent nanoprobes with good biocompatibility and high thermal sensitivity to obtain submicron spatial resolution and subdegree thermal resolution. Shi et al. [100] treated NIH-3T3 cells with NaYF_4_: Yb^3+^, Er^3+^@NaYF_4_: Yb^3+^, Nd^3+^ core–shell nanoparticles to measure the intracellular temperature (Figure 5).

Oxygen sensors have also been developed as the oxygen activity can be used for tumor diagnosis at the cellular level. Xu et al. [101] developed a multifunctional nanocomposite composed of light-emitting UCNPs capped with mesoporous silica and loaded with an oxygen-sensitive luminescent ruthenium complex. The red downconversion luminescence (at excitation/emission peaks of 455/606 nm) was rapidly reduced by oxygen and was able to rapidly indicate the oxygen concentration in hepatocellular carcinoma cells (HepG-2).

Such UCNP-based biosensors should be able to quickly and accurately measure changes in the specific parts of the cell. Therefore, SPT is indispensable in this field and accurate information that is collected at the cellular level by SPT analysis can be used in various important and specific applications, such as disease diagnosis.

## 4. Perspectives

We have briefly reviewed the principle of upconversion, usage of UCNPs in live cell imaging and SPT. SPT and imaging technology have been developed such that one can obtain nanometer (spatial) resolutions for imaging cells and tissues [27,29,32,40,102,103,104,105]. The bottleneck is more likely to be probes due to their photostability and targeting capability. Throughout this review, the advantages of using UCNPs as the probe were emphasized, but determining the specific targeting chemistry is still in its early stages. Medicinal chemists trying to find appropriate chemical drugs should be aware of the importance of imaging technology, such as SPT, and willing to incorporate it with this other technique. For example, photodynamic therapy requires sensitizing molecules attached to the nanoparticles and a laser system that triggers the reactive oxygen species (ROS) is produced [17,19,106]. A better understanding of the mechanism for the delivery of nanoparticles at the single particle level and chemical stability and reactivity is a prerequisite for early and accurate diagnosis and therapy [107,108,109].

## Figures and Tables

**Table 1:** Pros and cons of confocal, epifluorescence, and TIRF microscopy.

	Confocal	Epifluorescence	TIRF
Pros	No out-of-focus background Sectioning along *z*-axis	Rapid detection Low signal loss Deep penetration	Rapid detection Low background
Cons	Slow detection Signal loss	High background	Small excitation depth
